# Factors Associated with COVID-19 Vaccine Hesitancy among Visible Minority Groups from a Global Context: A Scoping Review

**DOI:** 10.3390/vaccines9121445

**Published:** 2021-12-07

**Authors:** Candy Ochieng, Sabrita Anand, George Mutwiri, Michael Szafron, Khrisha Alphonsus

**Affiliations:** School of Public Health, University of Saskatchewan, Saskatoon, SK S7N 5B5, Canada; candy.ochieng@usask.ca (C.O.); saa541@mail.usask.ca (S.A.); george.mutwiri@usask.ca (G.M.); michael.szafron@usask.ca (M.S.)

**Keywords:** vaccines, COVID-19, vaccine hesitancy, vaccine acceptance, visible minority, public health, vaccine concerns, vaccine intentions

## Abstract

Vaccine hesitancy is one of the top ten greatest threats to global health. During the COVID-19 era, vaccine hesitancy poses substantial risks, especially in visible minorities, who are disproportionately affected by the pandemic. Although evidence of vaccine hesitancy exists, there is minimal focus on visible minorities and the reasons for hesitancy in this group are unclear. Identifying these populations and their reasons for vaccine hesitancy is crucial in improving vaccine uptake and curbing the spread of COVID-19. This scoping review follows a modified version of the Arksey and O’Malley strategy. Using comprehensive search strategies, advanced searches were conducted on Medline, CINAHL, and PubMed databases to acquire relevant articles. Full-text reviews using inclusion and exclusion criteria were performed to extract themes of vaccine hesitancy. Themes were grouped into factors using thematic qualitative analysis and were objectively confirmed by principal component analysis (PCA). To complement both analyses, a word cloud of titles and abstracts for the final articles was generated. This study included 71 articles. Themes were grouped into 8 factors and the top 3 recurring factors were safety and effectiveness of the vaccine, mistrust, and socioeconomic characteristics. Shedding light on these factors could help mitigate health inequities and increase overall vaccine uptake worldwide through interventions and policies targeted at these factors. Ultimately, this would help achieve global herd immunity.

## 1. Introduction

Vaccine hesitancy is one of the top ten threats to global health and is defined as the “delay in acceptance or refusal of vaccines despite availability of vaccine services” [1]. It remains a major threat as it prevents the reduction of vaccine-preventable diseases [2]. Vaccination, a concept widely considered to be one of public health’s greatest achievements, currently prevents 2–3 million mortalities a year worldwide, and the World Health Organization (WHO) predicts that a further 1.5 million mortalities could be prevented by increasing the overall global coverage of vaccinations [2]. To reduce the prevalence and incidence of vaccine-preventable diseases, vaccination programs heavily rely on increased and sustained vaccine acceptance, which vaccine hesitancy and reluctance can impair [1]. Therefore, a thorough understanding and grasp of the factors that contribute to vaccine hesitancy and the interrelationships among one another are crucial in the development of effective public health vaccination campaigns and programs.

The WHO proposed a vaccine hesitancy model that is multifaceted, context-specific, and varies across time, place, and vaccines [3]. While the model categorizes factors influencing vaccine hesitancy into three broad categories: complacency, convenience, and confidence, because every individual has distinct lived experiences and principles, the factors influencing vaccine hesitancy may vary across different populations [3]. As vaccine hesitancy across various populations is a very dynamic concept, it becomes a major challenge during contingency situations like the COVID-19 global pandemic [4].

In this time of the global coronavirus disease, 2019 (COVID-19) pandemic with over 33 million people infected and about 1 million deaths worldwide [5], a significant portion of the public has been anticipating the development of an effective vaccine [6]. Mass vaccinations for COVID-19 are key in achieving herd immunity, however, vaccine hesitancy remains an obstacle to achieving herd immunity [7]. To overcome vaccine hesitancy, it is vital to identify populations that are likely to hesitate or resist COVID-19 vaccinations, as well as understand their reasons for doing so.

It has been noted that most research papers studying vaccine hesitancy have primarily focused on the general population consisting of different ethnicities for the results to be generalizable to the entire population while enhancing the quality of the study [8]. While some of these ethnicities have a high risk for both COVID-19 infection and COVID-19 vaccine hesitancy [3] and some vaccine hesitancy statistics do exist for some minority groups, there is a minimal focus in the literature on the reasons for vaccine hesitancy across visible minorities, as an entire group. Knowing factors influencing vaccine hesitancy in visible minorities in Canada is essential for dealing with the COVID-19 pandemic because, as per the Canadian 2016 census, almost 7.7 million Canadians identified themselves as a visible minority, accounting for nearly 22.3% of the total Canadian population [9]. Hence the purpose of this research is to identify reasons in the literature for COVID-19 vaccine hesitancy, specifically in visible minority populations, using an international perspective. We use an international lens in our review because many of the groups classified as visible minorities in Canada have immigrated to Canada [10].

## 2. Materials and Methods

Our working definition of visible minorities is as defined by The Employment Equity Act of Canada, which refers to visible minorities as “persons, other than Aboriginal peoples, who are non-Caucasian in race or non-white in color”. The visible minority population consists mainly of the following groups: South Asian, Chinese, Black, Filipino, Latin American, Arab, Southeast Asian, West Asian, Korean, and Japanese [3]. In addition to this, our working definition of the visible minority will incorporate the other designated groups of the Canadian Employment Equity Act; women, Aboriginal peoples, and persons with disabilities [3].

**Figure 1:**
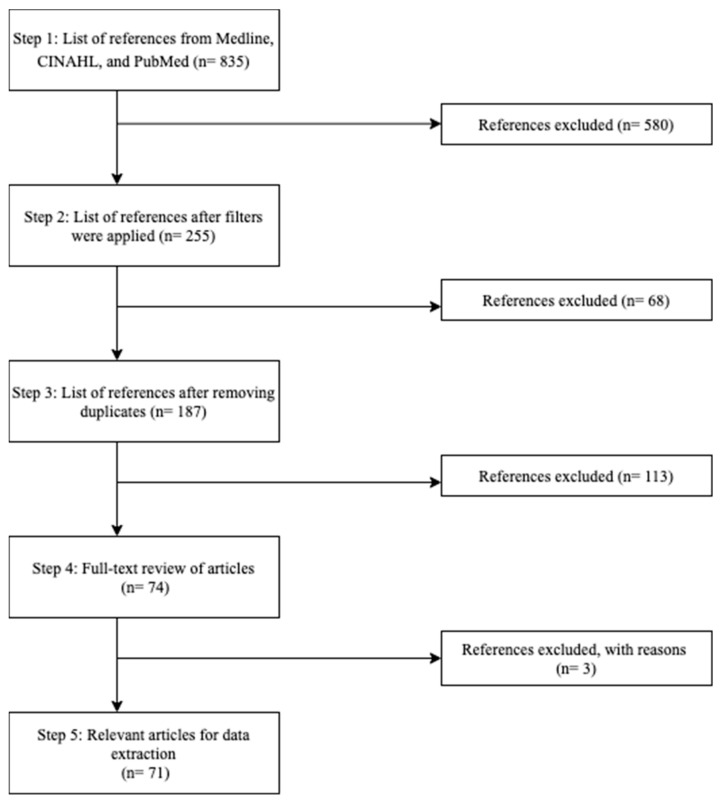
Flowchart depicting the steps taken to identify the relevant articles to be used for data extraction in our scoping review.

To explore our study objective, we conducted a scoping review following a modified version of the Arksey and O’Malley strategy [11]. Two reviewers conducted the scoping review to identify the key factors influencing COVID-19 vaccine decision-making in visible minorities. The specific steps we followed were as listed below:

1. We identified key concepts underlying our research question.
2. We identified search terms related to each key concept.
3. With the help of a public health librarian, we
  a. identified databases (Medline, CINAHL, and PubMed) from which to select literature; and
  b. created and implemented a search strategy for each database to generate a list of possible literature for review.
4. Two authors independently implemented the search strategies and identified literature from the three databases. They identified additional search terms from the literature and re-ran the searches. This was repeated until no new search terms were added to the search strategy.
5. These two authors independently implemented the final search strategy for each database to generate a list of possible articles for review. They then applied the filters: journal articles, full-text availability, papers published in 2020 and onwards, English language, and human participants, to create a reduced list of articles from each database.
6. A single reviewer transferred the reduced lists obtained in Step 5 to Zotero and compiled one list of articles for review while removing any duplicate articles.
7. Our scoping review process deviated from the Arksey and O’Malley strategy at this point because we hypothesized that findings regarding visible minorities and their respective reasons for vaccine hesitancy may not be found within the titles or abstracts of research papers. Therefore, to ensure that all relevant articles were included in our final list, we skipped title and abstract screening, and two reviewers performed full-text reviews applying the inclusion/exclusion criteria independently (Table 1) to create a final list of articles for data extraction. During this full-text review process, two reviewers discussed and resolved any disagreement that arose in terms of whether to include an article for data extraction. For when the two reviewers disagreed on whether or not to include an article, a third reviewer reviewed the article and decided on whether or not to include it. The final list of articles for data extraction was collated. The levels of agreement between both reviewers were calculated using the Cohen’s Kappa statistic.
8. While performing full-text reviews, the reviewers extracted themes of vaccine hesitancy in visible minority populations and stored them in an excel spreadsheet. Figure 1 depicts the steps taken to arrive at the final list of relevant articles for our scoping review.

Table 2 shows search terms used across all databases. The syntax was modified according to the requirements of each database. To maximize all the possible search concepts in all the articles from individual databases, we had to combine the three syntaxes to explore the full potential to ensure no more terms were left behind.

The syntax used in the three databases:

1. ADJ3: is a syntax used in a proximity search used in the Medline database—it is also known as (ADJ = adjacency). ADJ3 is the same as searching by having up to three words next to each other in any order. One needs to separate their search terms with ADJ3 in between when using this adjacency operator.
2. N3: is a syntax used in the CINAHL database, (N = near). N3 specifies there are three words between each search term while searching in no particular order.
3. AND, OR, NOT: are Boolean operators used in the PubMed database to retrieve all search terms results, retrieve results with at least search term or exclude search terms retrieved from the search.

### Data Analysis

To identify the key factors that influence COVID-19 vaccine hesitancy, three methodologies were performed:

1. Thematic qualitative analysis: Two reviewers independently grouped the extracted themes from the final list of articles that arose in step 8, into overarching factors. The level of agreement between both reviewers was calculated using the Cohen’s Kappa statistic.
2. Principal component analysis (PCA): Using the themes identified by both reviewers in step 8, a PCA with Promax oblique rotation was used to group the themes into overarching factors influencing COVID-19 vaccine hesitancy. A parallel analysis (with 1000 Monte Carlo simulation repetitions) was used to select the initial list of overarching factors. Themes that had a correlation of 0.6 or more or −0.6 or less with a factor were used to determine the name of the factor. An author different from the two who did the qualitative thematic analysis named the factors identified through the PCA.
3. Word cloud: To qualitatively complement the themes extracted during data extraction, a word cloud was created for 100 of the most frequently occurring words in the titles and abstracts of the final set of articles.

Following discussions among all authors of this research, the grouping of the themes into key factors identified by the PCA and word cloud were agreed upon. The Cohen’s Kappa statistic and PCA were computed using IBM SPSS Statistics 26.0. Word Cloud was generated using NVivo 12.

## 3. Results

In step 5, all three databases resulted in a total of 835 articles. Once the inclusion filters listed in step 5 were applied, the number of articles decreased to 255. In step 6, after duplicates were removed on Zotero, 187 articles remained. Captivatingly, our findings supported our hypothesis and all authors of this research agreed that a deeper search into the article must be performed rather than excluding articles based on the absence of relevant data in the title and abstracts. In step 7, upon full-text review of 187 articles, 113 were excluded due to falling within the exclusion criteria outlined in Table 2; 74 articles were retained in step 7. Within the 74 articles, three of them conflicted with the reviewers. After careful review, a consensus to exclude the three articles was made, which resulted in 71 relevant articles that fit the inclusion criteria and were ready for data extraction (Cohen’s Kappa statistic 0.966; *p* < 0.001). After the full-text review of all 71 articles, Table 3 lists 31 themes related to reasons for vaccine hesitancy that were identified in step 8.

A thematic qualitative analysis was performed to group the themes extracted from the literature into overarching factors, which resulted in the identification of 8 factors. A principal component analysis (PCA) was also a quantitative method of identifying key factors using the themes identified by both reviewers. While the PCA generated 16 factors with an eigenvalue greater than 1, the parallel analysis revealed 5 factors. As 8 factors were identified through thematic qualitative analysis, the 8 factors with the highest eigenvalues greater than 1 were extracted from the PCA (Table 4). The 8 factors identified by thematic analysis and the 8 factors identified by PCA were compared; 6 out of the 8 PCA factors were identical to 6 out of the 8 factors identified by thematic qualitative analysis (Table 5). The two factors that differed in PCA were convenience with getting vaccine and external factors while in the thematic qualitative analysis were vaccine development and perceived risk. After considering the remaining two factors identified by the PCA and the qualitative thematic analysis, by consensus, the authors decided to use the 8 factors suggested by thematic qualitative analysis.

**Figure 2:**
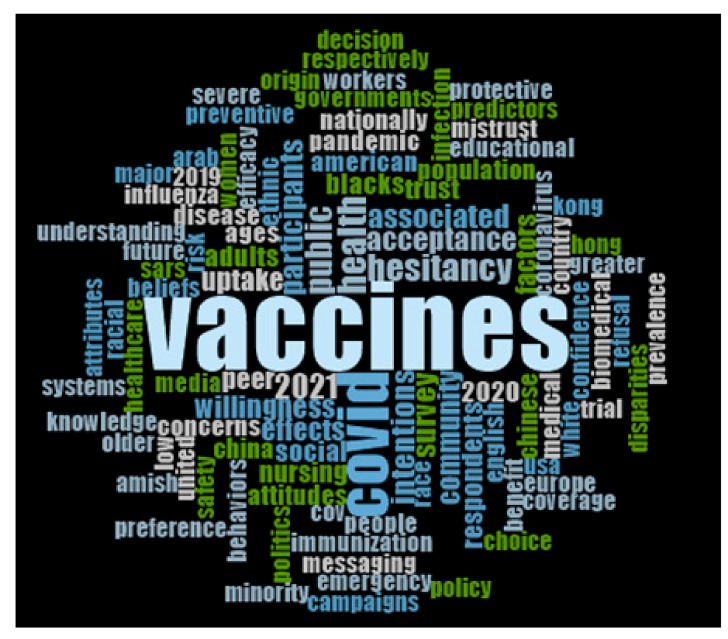
Word cloud displaying 100 of the most frequent words in the titles and abstracts of the 71 final set of articles.

The word cloud depicted 100 of the most frequent terms used in the titles and abstracts of the final 71 articles used in the scoping review. In the word cloud (Figure 2), terms such as “governments”, “women”, “influenza”, “beliefs”, “racial”, “knowledge”, “older”, “politics”, “mistrust”, “educational”, and “coverage” are presented, which complements the themes extracted during full-text review and the thematic qualitative analysis.

## 4. Discussion

In this study, we used two different approaches to identify overarching factors in the literature influencing vaccine hesitancy in visible minority populations: thematic qualitative analysis and principal component analysis (PCA). The 8 factors with the highest eigenvalues determined by the PCA were compared to the 8 factors identified by thematic qualitative analysis. Upon comparison, 6 out of 8 of the identified factors matched, including the three most frequently discussed factors. The most frequent factor that appeared in the literature was safety and effectiveness of the vaccine with themes classified under this factor being extracted 88 times in total. The second most frequently discussed factor was mistrust with themes classified under this factor being extracted a total of 72 times, followed by socioeconomic characteristics of the people with themes related to this factor being extracted 52 times (for the frequencies associated with all 8 factors, refer to Table 3).

Our analyses identified concerns around the safety and efficacy of the COVID-19 vaccine as the most frequently appearing reason in the literature for COVID-19 vaccine hesitancy. The themes from the literature that were classified within this factor were safety, vaccine effectiveness, short-term and long-term side effects, fear of needles, and the number of injections. With respect to side effects, people of Muslim religion expressed COVID-19 vaccine hesitancy because of concerns related to how the side effects associated with the COVID-19 vaccine may interfere with the Festival of Ramadan [12]. In a study involving Black and Latinx long-term care staff, both ethnicities reported the possibility of developing side effects because of vaccination as their primary reason for being vaccine-hesitant [13]. In a UK-based study involving ethnic minorities, the most common reasons for hesitancy included side effects and long-term health effects, particularly among Black respondents [14]. Short-term side effects of concern that were commonly reported varied from prolonged injection site pain across people of African American descent [15]. On the other hand, more long-term health concerns were centered around fertility issues for women, specifically in the Arab female population [16].

In a study involving Black persons living with HIV, half of the participants perceived the COVID-19 vaccine as harmful and were worried about the safety of the vaccine [17]. Similar findings were obtained in Filipino populations, where nearly half were opposed to receiving the vaccine due to safety concerns [18]. Apprehension towards the safety of the COVID-19 vaccine differed across race and ethnicity, with most Black participants (66%) citing this as a reason for refusing the vaccine, followed by Hispanics (47%) and others (14%) [19]. Shifting to Indigenous populations in Canada, vaccine hesitancy remains a significant challenge compared to other populations within Canada [20]. Fear and concerns around the safety of the COVID-19 vaccines arise from medical experimentations that took place using Indigenous peoples to test the safety and effectiveness of vaccines [20,21]. Kreps et al. found that women and Black respondents were less likely to report willingness to take the vaccine especially due to concerns surrounding vaccine efficacy and the possibility of severe vaccine adverse effects [22].

The frequency of injections and vaccine schedules influenced the decision-making of certain visible minorities. Female respondents were more likely than male participants to report a higher willingness to vaccinate if the vaccine involved fewer injections [23]. On a similar note, a study conducted in the USA indicated that among racial and ethnic groups, non-Hispanic Blacks were least likely to vaccinate due the perception of the complicated vaccine schedule [24]. Several reasons underly how the frequency of injections/vaccine schedules impact vaccine hesitancy. Respondents preferred fewer doses, partly because fewer visits to immunization sites save transportation and time costs [25]. For those who fear needles, as the number of injections required increased, the chances of receiving vaccinations voluntarily decreased [25].

Our analyses identified mistrust as the second most-frequent factor appearing in the literature regarding COVID-19 vaccine hesitancy in visible minority populations. The themes linked to this factor include pharmaceutical/government medical mistrust, racism, underrepresentation in medical clinical research, and biased non-diverse healthcare providers. Mistrust was used as a broad term to explain three types of mistrust: medical mistrust, government mistrust, and pharmaceutical mistrust. According to Thompson et al., the Middle East and North Africa (MENA) populations in the USA are underrepresented in health inequity because the US government does not recognize them as a minority group that is distinct from the white ethnicity [26]. This minority group experiences healthcare barriers due to medical mistrust and discrimination [26]. The participants in the Bogart et al. study showed high rates of COVID-19 mistrust and hesitancy related to future vaccines [17]. Nearly all (97%) Black participants validated one mistrust belief, with the most being mistrust due to the government withholding of information concerning COVID-19 [17]. This mistrust of Black Americans can be directly linked to events in the course of medical history in the United States, such as the Tuskegee study [27]. Another study by Laurencin et al. elaborates how Black ethnicity has been affected by mass incarceration, poverty, and limited healthcare access [28]. These factors are linked to racism and segregation that has been part of the Black community for decades, which has created increased numbers in COVID-19 vaccine hesitancy today [28]. Another example of racism’s impact on COVID-19 vaccine hesitancy is that due to racism, non-Hispanic Blacks were least likely to get a COVID-19 vaccine [24].

The lack of visible minority representation in the medical profession has a profound impact on COVID-19 vaccine hesitancy. For example, Black men are less represented in the medical profession: while they comprise about 13% of the US population, they only comprise 4% of US doctors, and less than 7% of the US medical students [28]. Vulnerable populations have a difficult time trusting the medical profession; this can be mitigated if the healthcare providers come from their own communities [29]. Laurencin et al. show an increase in the number of black men in the healthcare profession would increase the trust in the Black community on matters concerning COVID-19 vaccination [28]. Further trust can be earned if there is an adequate representation of racial minority groups in vaccine trials [30].

Socioeconomic characteristics were the third most frequent factor in the literature for visible minority populations being COVID-19 vaccine-hesitant. The themes from the literature categorized in this factor include gender, age, education, income, occupation, location, and having children. Several studies have investigated how themes influence an individual getting a COVID-19 vaccine. Women in the USA were more likely to refuse the vaccine because they tend to practice preventive behaviors and avoid risky behaviors, e.g., wearing face masks to prevent COVID-19 infections [31]. In the USA, support towards vaccination for COVID-19 increased with age across Black Americans and other populations [32]. Education plays a significant role; in a Canadian study, learned individuals with less than a high school education showed lower adjusted odds of wanting to vaccinate themselves against COVID-19 [33]. Furthermore, Black Americans with lower educational attainment are more hesitant to accept a COVID-19 vaccine [32]. A study conducted in Latin America and the Caribbean disclosed similar findings but added that lower education influences vaccine hesitancy due to the general distrust in vaccines and the robustness of conspiracy beliefs across individuals with lower education levels [34]. In terms of occupation, essential Canadian non-healthcare workers were shown to have lower odds of intending to receive the COVID-19 vaccine when it is available [33]. In the USA, research by Allen et al. found that ethnic minorities such as Chinese, Black, Latina, and others were less likely to report the intention to vaccinate with lower levels of income [30]. Similar findings by Nikolovski et al. and Khubchandani et al. were observed in African American individuals located in the USA [15,31]. In an Ohio-based Amish population, one reason for vaccine refusal was that those who had children believed that if they gave their children shots, it would imply that they were not putting their faith in God to look after their children [35]; hence families with children were more likely to refuse COVID-19 vaccination [35].

Vaccine development was the fourth most common factor in the literature influencing COVID-19 vaccine hesitancy in visible minority populations. Themes that fell under this factor included the place of manufacturing for the vaccine, cost of the vaccine, the vaccine’s novelty, frequency of injections associated, short duration of development, and the duration of immunity. As for the place of vaccine manufacturing, in a study by Gramacho et al., one factor that has a higher chance of increased vaccine uptake was the concerns on where the vaccine was manufactured and developed [36]. Respondents from a study conducted by Kreps et al. were less likely to choose vaccines developed outside of the United States, particularly from China, the associated vaccines manufactured outside the USA with a lower probability of choosing the vaccine [37]. In addition, rushed vaccines developed under a presidential administration with less transparency to the consumers were a recipe for suspicion, regardless of ethnic background [38]. COVID-19′s novel vaccination technologies, utilizing the messenger RNA and adenoviral transgene delivery, not previously used in the general population, were associated with the generation of many questions that would need answers to help clear the uncertainty among the people [29]. In terms of the cost of the vaccine, the COVID-19 vaccine price being high in the market may deter a substantial share of the at-risk people, especially those earning low incomes, from getting vaccinated against COVID-19 [39]. Furthermore, another study by Gatwood et al. suggested that Hispanics, in comparison to Whites and Blacks, had a higher agreement with the statement “New vaccines carry more risks than older vaccines” [32]. A study by Green et al. showed that Arab respondents felt that childhood vaccines differ from the COVID-19 vaccines because childhood vaccines have been well-integrated into the healthcare system, whereas COVID-19 are very new [16]. A study conducted in the USA indicated that among racial and ethnic groups, non-Hispanic Blacks were least likely to vaccinate due to lack of confidence in the vaccine [24]. When it comes to the short duration of vaccine development, according to Thompson et al., vaccine uptake rejection among Black participants compared with the overall mean rejection was flagged as one of the reasons why the Black ethnicity was hesitant to get the COVID-19 vaccine [26]. According to a study by Khubchandani et al., high vaccine hesitancy could be linked by a response from the participants in a Kaiser Family Foundation (KFF) poll where a majority (62%) believed that sociopolitical factors and pressures lead to a rushed approval for the COVID vaccine [31]. Finally, the duration of immunity that the COVID-19 vaccine would provide plays a role in influencing vaccine uptake. A study involving Black and Latinx participants revealed that among several factors that would matter in their vaccination decisions was how long protection from the vaccine lasts, with nearly 68% of participants supporting that [40]. Similarly, a longer duration of immunity of 5 years in comparison to 1 year was associated with higher support towards receiving the COVID-19 vaccine [22].

The fifth factor identified was information circulation. Themes in this factor included the type of media information, information reliability, and political ideology. In a study by Latkin et al., lack of public trust in the CDC, with unreliable messages on the approaches used for COVID-19 testing and testing delays were linked with increased numbers in COVID-19 vaccine hesitancy [19]. The type of information circulating on social media platforms has also played a vital role in increased numbers on vaccine hesitancy towards the COVID-19 vaccine. A study by Nikovolski et al. [15] on the type of media information themes identified the potential for vaccine-related data to shift the perception of individuals. For example, Nikovolski et al. [15] found that negative stories on vaccine adverse effects circulating online were associated with an increased unwillingness for people to get vaccinated for COVID-19. The study [41] showed how information reliability resulted in some Arab ethnic community members believing that the COVID-19 vaccine contained non-Halal or alcohol-based components, thus in turn negatively affecting the COVID-19 vaccine uptake. A study by Gatwood et al., on matters concerning how political ideology impacts COVID-19 vaccine hesitancy, states COVID-19 vaccine hesitancy was more likely among those with more moderate or conservative political leanings, Black Americans and, residents of nonmetropolitan areas [32]. Black participants trusted the healthcare system and a president of a conservative party less, all due to the systemic racism they have experienced for decades [42]. Moreover, they were less likely to be influenced to change their negative perspective on their COVID-19 vaccine-related beliefs, compared to the rest of the study sample [42]. A study conducted in Brazil elaborates how the support of political members has a direct influence on the increased vaccine uptake. Supporters of a certain Brazilian president were likely to vaccinate at a higher rate as compared to respondents who were less supportive of him [36].

Knowledge and acceptance of vaccines was the sixth most common factor in the literature influencing COVID-19 vaccine hesitancy in visible minority populations. This factor was comprised of general beliefs towards vaccines, past vaccine compliance, and religious beliefs that play a role in affecting COVID-19 vaccine acceptance. With regard to reluctance towards vaccination in general, research by Allen et al. studied Chinese, Black, Latina, and other populations, revealing that nearly 12% of respondents declined vaccination or were unsure because they were generally distrustful of vaccines [30]. Disparate intentions were observed across people of the Black race, particularly, due to the less favorable beliefs about vaccines, which reflect higher hesitancy in this population [42]. Some American-Amish, a notable minority group, believed that it is better to have natural immunity instead of vaccines, therefore, their beliefs towards vaccines, in general, is a strong influencer of vaccine hesitancy [35]. Past vaccine compliance towards other vaccines, such as the yearly Influenza vaccine, influences whether an individual was intending to receive the COVID-19 vaccine. Wang et al. found that previous influenza vaccination behavior was strongly indicative of an intention to vaccinate against COVID-19 [43]. Similar findings were obtained across Black and Hispanic people who have historically been less likely to receive Influenza vaccines [13]. Lastly, within this factor lies religious beliefs. Among Amish populations, those who rejected vaccines stated that for them, a religious leader was the most influential person in determining whether to take the vaccine; therefore for a portion of the Amish community, vaccine decisions are significantly impacted by religion [35]. In addition to this, festivals like Ramadan require Muslims to refrain from consuming food drinks during daylight; it also requires abstaining from anything entering the body cavities [12]. Hence during the Ramadan fasting hours, several people were reluctant to receive the COVID-19 vaccine [12]. Hesitancy among several Muslim and other religious groups was also observed because of the perception that pork gelatine was one of the ingredients in the COVID-19 vaccine [44]. Among Arabs and ethnic minorities in a study conducted in Israel, UK, and the US, it was found that along with family and friends, religious leaders are relatively high influencers in vaccine decision-making [41].

The seventh factor identified from the literature influencing COVID-19 vaccine hesitancy in visible minority populations was the perceived risk of COVID-19. Themes underlying this factor included the perceived risk of acquiring COVID-19 infection as well as a health care professional’s opinion on receiving the vaccine. A USA-based study found that the perceived threat of COVID-19 is a strong predictor of vaccine hesitancy across Black, Asian, Hispanic, and other ethnicities [31]. The ability to assess this perceived threat depends on the level of awareness [31]. The medical opinion of a health care professional also can influence whether an individual chooses to vaccinate themself. For example, a study using African American participants found that of those not willing to receive a vaccine, over 75% of them preferred to take their health care professional’s opinion first [15] and if a doctor recommended the vaccine [40]. Another study found that more than half the African Americans in the study were less likely to rely on their healthcare provider for COVID-19 vaccine information [45] and had the lowest level of agreement with the statement “generally I do what my healthcare provider recommends about vaccines” [32].

The final factor our thematic analysis identified was perceived benefit, which includes the number of people vaccinated, collective and personal benefit themes. Some individuals of different racial and ethnic backgrounds rely on the number of people surrounding them that have received the vaccination to determine if they will receive the vaccine or not [25]. In terms of personal and collective benefit, a study conducted by [46] showed significant differences by ethnicity, with Asian individuals showing the greatest reduction in hesitancy in comparison to Black ethnicity [46]. Individuals of Black ethnicity sometimes reacted negatively towards information on collective and personal benefits of taking the COVID-19 vaccine. In particular, information regarding the collective benefit of not transmitting COVID-19 rendered these individuals more reluctant to receive the vaccine [46]. In Gatwood et al., Black persons showed the least agreement with the statements “being vaccinated is important for the health of others in my community” (collective benefit) and “getting vaccines is a good way to protect me from the disease (personal benefit) [32].

A study on ethnic differences in SARS-CoV-2 vaccine hesitancy in United Kingdom healthcare workers provides the percentage of vaccine hesitancy among different populations: Black Caribbean (54.2%), Black African (34.4%), Chinese (33.1%), and Pakistani (30.4%) while amongst the White British healthcare workers (21.3%) [47]. We correspondingly suspect that percentages of COVID-19 vaccine hesitancy will also differ by visible minority group. Hence, we also suspect that the impact of our identified factors for COVID-19 vaccine hesitancy among visible minority groups will differ by group.

Overall, rates of vaccine acceptance varies between different cultural and geographical contexts [48]. A systematic review conducted by Cascini et al. found that Arabian countries have shown to have higher reported vaccine hesitancy compared to other nations of the world [48]. Similarly, although China has higher COVID-19 vaccine acceptance rates as opposed to other countries such as France and the US, a study by Qin et al. stated that older adults are less willing to receive a COVID-19 vaccine [39]. This high-risk population was also found to not be willing to pay as much for the vaccine, compared to the general population [39]. This was further supported by a Chinese national cross-sectional study finding of 35.5% being vaccine hesitant [49]. Thus, differences in vaccine reluctance not only exist between countries of the world, but also exist within a country. On that note, it is important to not generalize the factors influencing COVID-19 vaccine hesitancy in one minority group to their entire country. For example, vaccine hesitancy factors that play a role in some Chinese populations should not be generalized to the entire population of China.

## 5. Conclusions

A thematic qualitative analysis identified 8 factors in the literature for COVID-19 vaccine hesitancy in visible minority populations. Safety and effectiveness of the vaccine, mistrust, and socioeconomic characteristics were the most frequently occurring factors across the 71 articles identified by our scoping review. A reality of this small number of identified studies is visible minority groups are underrepresented in COVID-19 vaccine hesitancy researcher. Knowing how different factors influence COVID-19 vaccine hesitancy in visible minority populations is essential for developing customized strategies for improving vaccination rates in different minority populations. While we present our identified factors for COVID-19 vaccine hesitancy in visible minority populations as distinct factors, each factor does not impact vaccine hesitancy in isolation. Understanding the relationships among our identified factors and understanding how our factors impact each visible minority group are essential to improving the health of visible minority groups while helping our global population reach COVID-19 herd immunity thresholds.

## Figures and Tables

**Table 1:** Inclusion and exclusion criteria.

Inclusion	Exclusion
Peer-reviewed journal articles	Non-peer reviewed articles/grey literature
Articles related to COVID-19 and visible minorities	Articles studying diseases other than COVID-19 and non-visible minorities
Papers written in English language	Papers written in languages other than English language
Papers published in 2020 and onwards	Papers published prior to 2020
Papers involving human participants	Papers involving non-human participants
Articles discussing reasons for COVID-19 vaccine hesitancy	Articles not discussing reasons but rather stating statistics related to COVID-19 vaccine hesitancy
	Articles studying COVID-19 vaccine hesitancy in clinical/vaccine trial participation

**Table 2:** Search concepts used in the Medline, PubMed, and CINAHL databases.

Concept 1 (Vaccine): (Combine Terms below with OR)	ADJ3 *	Concept 2 (Hesitancy): (Combine Terms below with OR)	AND	Concept 3 (Visible Minority): (Combine Terms below with OR)	AND	Concept 4 (COVID-19): (Combine Terms below with OR)
Vaccin * Inoculat * Immuniz * Inject * Shot Shots		Hesita * Confiden * Refus * Decision Decisions Decision making Concern * Accept * Attitud * Barrier Barriers Trust * Adher * Uptak * Reluctan * Skeptic * Intent *		Visible minorit * (Race OR racial OR ethnic) ADJ3 (disparit * OR inequality * OR inequit * OR segregate * OR minorit *) Ethnic * Divers * Vulnerable * (Vulnerab * ADJ3 population *) Non-white * Non-Caucasian * (South ADJ3 Asian *) Chinese (Black OR Blacks OR African ADJ3 American *) Filipino * (Latin American * OR Latino * OR Hispanic *) Arab * (Southeast ADJ3 Asian *) (West ADJ3 Asian *) Korean * Japanese Aboriginal * Indigenous Native * First Nation * Indian Indians Metis Inuit		COVID-19 COVID-19 Coronavirus Corona virus Coronavirus 19 SARS CoV 2 Coronavirus disease 2019 COVID 2019 COVID19 Global pandemic Novel coronavirus Novel corona virus 2019 nCoV nCoV 2019 2019 ncov CoV 2019 2019 CoV ncov19 ncov 19 2019 novel CoV SARS coronavirus 2 Sars coronavirus 2 SARS like coronavirus Severe Acute Respiratory Syndrome Coronavirus 2 Severe acture respiratory syndrome coronavirus 2 severe-acute-respiratory-syndrome-coronavirus-2 wuhan OR hubei OR huanan OR china OR chinese) AND (severe acute respiratory OR pneumonia *) AND outbreak *) cov-19 COVID pandemic Coronavirus infection Coronavirus infections

* Indicates wild card for search term endings.

**Table 3:** Factors, their underlying themes, and the number of articles containing the factors/themes as extracted from the final 71 articles.

Factor	Themes	Frequency
**Safety and effectiveness of vaccine**		**88**
	Safety	31
	Effectiveness	31
	Side Effects	22
	Frequency of Injections/Fear of Needles	4
**Mistrust**		**72**
	Lack of Trust	39
	Racism	21
	Underrepresentation in Medical/Clinical Research	10
	Biased/Non-diverse HCPs	2
**Socioeconomic characteristics**		**52**
	Gender	21
	Age	12
	Education	9
	Income	5
	Occupation	2
	Location	2
	Having children	1
**Vaccine development**		**45**
	Short duration of Vaccine Development	14
	Cost of Vaccine	12
	Place of Manufacturing	8
	Novelty of Vaccine	7
	Duration of Immunity	4
**Information circulation**		**42**
	Information Reliability	18
	Type of media Information	17
	Political Ideology	7
**Knowledge and acceptance**		**18**
	Religious Beliefs	8
	General Vaccine Beliefs	7
	Past Vaccine Compliance	3
**Perceived risk of COVID-19**		**17**
	Perceived risk of acquiring COVID-19	13
	infection	
	Opinion of HCP	4
**Perceived benefit**		**5**
	Personal Benefit	2
	Collective Benefit	2
	Number of people Vaccinated	1

**Table 4:** Key factors, their underlying themes, and their associated loadings resulting from the PCA.

Factor (Eigenvalue)	Themes (Loading)
Perceived benefit (4.584)	Occupation (0.688)
	Collective benefit (0.916)
	Personal benefit (0.916)
Safety and effectiveness of vaccine (3.532)	Side effects (0.714)
	Safety (0.820)
	Effectiveness (0.862)
Socioeconomic characteristics (2.863)	Income (0.834)
	Location (0.785)
	Having children (0.767)
Convenience associated with getting the vaccine (2.525)	Frequency of injections/schedule (0.656)
	Location (0.656)
	Number of people vaccinated (0.855)
Knowledge and acceptance of vaccine (2.099)	Education (0.622)
	Past vaccine compliance (0.699)
	General vaccine beliefs (0.665)
Mistrust (2.002)	Lack of trust (0.787)
Source of information about vaccine (1.855)	Type of media information
	Information reliability
External factors (1.706)	Duration of immunity (0.623)
	Opinion of HCP (0.643)
	Pre-existing medical conditions (0.619)
	Vaccine coverage (0.794)

**Table 5:** Factors in agreement from thematic qualitative analysis and principal component analysis.

Factors from Thematic Qualitative Analysis	Factors from Principal Component Analysis	Factors in Agreement
Perceived benefit	Perceived benefit	Perceived benefit
Safety and effectiveness of vaccine	Safety and effectiveness of vaccine	Safety and effectiveness of vaccine
Socioeconomic characteristics	Socioeconomic characteristics	Socioeconomic characteristics
Information circulation	Source of information about vaccine	Source of information about vaccine
Knowledge and acceptance	Knowledge and acceptance of vaccine	Knowledge and acceptance of vaccine
Mistrust	Mistrust	Mistrust
Vaccine development	Convenience associated with getting the vaccine	
Perceived risk of COVID-19	External factors	

## Data Availability

Data sharing not applicable.
